# Supplementary material for: Prevalence and factors associated with Intestinal parasitosis among adults with diabetes mellitus attending a tertiary care facility in Northern Uganda: A hospital-based cross-sectional study
Source: PLoS One. 2025 Dec 26;20(12):e0338774. doi: 10.1371/journal.pone.0338774 (PMC12742753; doi:10.1371/journal.pone.0338774)
Supplement: S1 File — This is the questionnaire in English. (PDF) [file pone.0338774.s001.pdf]

APPENDIX II: QUESTIONNAIRE

Version 2.0

Participant ID: ……………………………

SECTION 1: SOCIO DEMOGRAPHIC DATA

1 What is your age?

………………………………………………

2. Gender
   A. Male ☐
   B. Female ☐

3. Tribe
   ☐ Acholi
   ☐ Langi
   ☐ Madi
   Others (Specify) …………………………………………………

4. Marital Status
   ☐ Single
   ☐ Married
   ☐ Divorced
   ☐ Widowed

5. Education Level
   ☐Primary
   ☐Secondary
   ☐Tertiary
   ☐No Formal Education

6. Residence
   A. Rural ☐
   B. Urban ☐

7. Occupation
   ☐ Government Employee
   ☐ Farmer
   ☐ Student
   Other (Specify) ……………………………………………

8. Religion
   ☐ Anglican
   ☐ Catholic

☐ Moslem
Other (Specify) ……………………………………..

SECTION 2: CLINICAL CHARACTERISTICS

9. When were you diagnosed with Diabetes Mellitus?
………………………………………………………..

10. Type of Diabetes Mellitus
    A. Type 1 ☐
    B. Type 2 ☐

11. Blood Sugar Control
    A. Well controlled ☐
    B. Poorly controlled ☐

12. Diabetic complications
    A. YES ☐
    B. NO ☐

13. If yes in 12 above, select from among the following
☐ Neuropathy
☐ Retinopathy
☐ Nephropathy
Others(Specify) ……………………………………….

14. Current Treatment
☐ Oral
☐ Insulin
☐ Both
☐ None

15. Are there comorbidities?
    A. YES ☐
    B. NO ☐

16. If yes in 15 above, select among the following?
☐ Hypertension
☐ Heart Disease
☐ Cancer
Others (Specify) ……………………………………………………

17. Have you ever been diagnosed with Intestinal Parasites?
    A. YES ☐
    B. NO ☐

18. If yes in 17 above, specify the type
    A. Ascaris Lumbricoides ☐

B. Hook worms ☐

C. Trichuris Trichuria ☐

D. Strongyloides Stercolaris ☐

E. *Others (Specify)………………..*

19. Have you received treatment for Intestinal Parasites?

A. YES (Specify) ……………………………………………………………..

B. NO ☐

20. Do you have any family member diagnosed with Intestinal Parasites?

A. YES ☐

B. NO ☐

SECTION 3: LIFE STYLE AND ENVIRONMENTAL FACTORS

21. Do you use footwear when walking outdoors?

A. Rarely ☐

B. Often ☐

C. Never ☐

22. Do you have domestic animals at home?

A. YES ☐

B. NO ☐

23. Do you wash your hands before eating?

☐ Always

☐ Sometimes

☐ Never

☐ Rarely

24. What is your water source?

☐ Borehole

☐ Tap water

☐ River/Stream

Others(Specify) ……………………………………………….

25. How do you dispose off human wastes?

☐ Pit Latrine

☐ Toilet

☐ VIP latrine

Others (Specify) ………………………………………………

26. Have you experienced any of the following symptoms?

A. Abdominal pain ☐

B. Diarrhea ☐

C. Nausea/Vomiting ☐

D. Weight Loss ☐

E. Others (Specify) ………………………………………

27. Have you sought medical attention for any of these symptoms

A. YES ☐

B. NO ☐

28. Were those symptoms linked to Intestinal Parasites based on medical diagnosis?

☐ YES

☐ NO

☐ NOT SURE

SECTION 4: LABORATORY RESULTS

29. Type of Intestinal Parasites diagnosed from stool

A. *Hook worms* ☐

B. *Ascaris lumbricoides* ☐

C. *Strongyloides stercolaris* ☐

D. *Trichuris trichiuria* ☐

E. *Others (Specify)……………………………………..*
