# Supplementary material for: Prevalence and factors associated with Intestinal parasitosis among adults with diabetes mellitus attending a tertiary care facility in Northern Uganda: A hospital-based cross-sectional study
Source: PLoS One. 2025 Dec 26;20(12):e0338774. doi: 10.1371/journal.pone.0338774 (PMC12742753; doi:10.1371/journal.pone.0338774)
Supplement: Attachment — Submitted filename: Response to Reviewers.docx [file pone.0338774.s003.docx]

**RESPONSE TO REVIEWERS**

On behalf of the authors’ team for our study “*Prevalence and factors associated with Soil-Transmitted Helminths Infections among adults with diabetes mellitus attending a tertiary care facility in Northern Uganda: A hospital-based cross-sectional study*”, we sincerely thank the reviewers for the insightful and constructive feedback on our manuscript. The comments we received have significantly strengthened the clarity, rigor, and impact of our work. In the table below, we provided a point-by-point response to each comment we received, explaining the revisions made and indicating where the changes were incorporated in the revised manuscript. We believe these changes address all concerns and enhance the paper’s contribution to the field. We look forward to your kind feedback.

**Table showing response to reviewers’ comments**

| **S/No** | **COMMENT** | **RESPONSE** | **PAGE Number (As in track change file)** |
| --- | --- | --- | --- |
| **ACADEMIC EDITOR’S COMMENTS** | | | |
| 1 | The authors should reframe the title and abstract to reflect the broader focus on intestinal parasitosis | The title and abstract have been reframed | Page 1 and 2 |
| 2. | Provide a more robust justification for the sampling strategy | We used convenience sampling technique because;  Most of the patients attending the clinic were adults, and a number of them were not willing to provide a stool sample for the study, claiming that it was uncomfortable and that they had no urge to pass stool.  Additionally, we had limited resources to sustain a probability sampling technique, given the limited time we had for data collection.  In light of the above reasons, convenient sampling technique was adapted. | Pg 7 |
| 3 | transparently address methodological limitations such as single stool sampling and lack of molecular diagnostics | We collected a single stool sample because we only had the chance to interface with participants as they came for their routine Diabetic care review, which happens at a 1-month interval, and we could therefore not access them easily to provide the 3 separate samples within a 2 to 3 day interval as recommended by WHO.  Also, our participants were from distant places and could not come to provide stool samples on 3 separate days, due to transportation constraints.  We also had logistical constraints to support the laboratory work for the 3 samples for each patient | Pg 24 |
| 4. | Additionally, the ethical approval timeline | We received initial approval on 24/09/2024 and started data collection in October 2024. We then made amendments, and the amendment approval was given on 30/05/2025 | Pg 8 |
| 5. | Data availability statement must be clarified and harmonized to meet journal standards | We have addressed this by emphasizing that; All relevant data are within the manuscript and its supporting information files. Data are available upon reasonable request from the first author | Pg 27 |
| **REVIEWER 1 COMMENTS** | | | |
| 1. | Zero STHI prevalence is a major finding but is not sufficiently contextualized within national deworming or public health programs that may explain this result. | We have considered intestinal parasitosis instead of STHIs, as guided by the academic editor.  This change has been embraced throughout the manuscript. |  |
| 2. | The title and abstract emphasize STHIs, yet the study found non this creates a mismatch between stated objectives and actual findings. | The title and abstract have been modified to emphasize intestinal parasitosis, and the objectives and findings have also been restructured in line with intestinal parasitosis | Title page, Pg 1,2, |
| 3. | Reframe the study focus: Given the absence of STHIs, consider repositioning the paper to emphasize intestinal parasitosis broadly among DM patients in Uganda. | We have reframed the study to focus on intestinal parasitosis | Throughout the manuscript |
| 4. | Sample size justification uses Slovin’s formula, which is simplistic and not ideal for prevalence studies; a more robust epidemiological formula (e.g., Cochran’s) would be preferable. | We've adjusted to use the Cochran formula, which gives us a sample size of 267 participants and a response rate of 75.3% (n=201/267) | Pg 7 |
| 5. | Convenience sampling introduces selection bias and limits generalizability; this limitation is underemphasized. | We have included this limitation in our limitations section | Pg 24 |
| 6. | Only one stool sample per participant was analyzed, which significantly reduces sensitivity for detecting STHIs due to intermittent egg shedding. | This was because we only had the chance to interface with participants as they came for their routine Diabetic care review, which happens at a 1-month interval, and we could therefore not access them easily to provide the 3 separate samples within a 2 to 3 day interval as recommended by WHO.  Also, our participants were from distant places and could not come to provide stool samples on 3 separate days, due to transportation constraints.  We also had logistical constraints to support the laboratory work for the 3 samples for each patient | Pg 24 |
| 7. | Given that no STHIs were detected, how do you reconcile this with Uganda’s reported STHI prevalence of 5–50% in other regions? | We have reframed our manuscript to focus on intestinal parasites rather than STHIs.  With the reframing, our study now emphasizes the presence of intestinal parasitosis, at a prevalence of 19.4% unlike the initial absence of STHIs. | Pg 16 |
| 8. | The methods section lacks detail on how the Kato-Katz and Odongo-Aginya methods were specifically applied (e.g., number of slides, timing, technician training). | This has been addressed. Two thick smears were prepared, and analysis was done within a 4-hour interval from the time of sample collection. The stool analysis was performed by 1 laboratory technician and 1 professor of parasitology. | Pg 7 |
| 9. | No molecular diagnostics (e.g., PCR) were used, despite their known higher sensitivity for helminths like Strongyloides stercoralis. | This was not possible due to limited logistics. No PCR tests were being done in the laboratory that analyzed our samples, and additionally, we had limited funds to support analysis elsewhere. | Pg 24 |
| 10. | The discussion does not adequately explore why STHIs were absent, especially given Uganda’s reported 5–50% STHI prevalence. | The discussion has been reframed to focus on intestinal parasitosis and not STHIs, since they were absent | Pg 19-23 |
| 11. | Entamoeba coli is mischaracterized as “non-pathogenic normal flora,” while generally non-pathogenic, its presence indicates fecal-oral contamination and poor sanitation. | We have re-characterized it under pathogenic parasites and discussed the need to embrace the WASH strategy to mitigate its transmission, since its presence indicates ongoing fecal-oral contamination. | Abstract and Pg 19 |
| 12. | Include a limitation paragraph explicitly stating that the inability to perform association analyses due to zero STHI cases is a major constraint. | Since we’ve reframed our manuscript to focus on intestinal parasitosis, and we found intestinal parasitosis in 19.4% of the participants, we have performed association analyses | Pg 17-19 |
| 13. | Candida albicans is mentioned incidentally but without a clinical context. Was this considered colonization or infection? | It was considered a colonization |  |
| 14. | Statistical analysis of associated factors is missing despite the aim to identify “factors associated with STHIs,” no inferential analysis could be performed due to zero cases, yet this is not explicitly acknowledged. | We’ve reframed our manuscript to focus on intestinal parasitosis, and since we found intestinal parasitosis, inferential analysis has been performed | Pg 17-19 |
| 15. | Was seasonality accounted for? The study ran from October 2024 to March 2025. Could dry vs. rainy seasons affect STHI transmission? | Yes, seasons affect STHI transmission, with the transmission being higher during rainy seasons due to increased contamination from running water and latrine overflows, and favorable conditions of breeding for the vectors.  The wet season in our study area ranges from April to November, and the dry season ranges from November to March.  However, we couldn't halt data collection until the wet season begins, as we would not meet the timeline that had been given to us to conduct the study. |  |
| 16. | Ethics approval date discrepancy: Approval was granted in 2024, but an amendment is dated 30/05/2025, which is in the future relative to the current date (October 2025). This needs clarification. | We received initial approval on 29/09/2024 and started data collection in October 2024. We then made amendments, and the amendment approval was given on 30/05/2025. | Pg 8 |
| 17. | Inconsistent data availability statement: The submission form states “All relevant data are within the manuscript,” but the manuscript says “Data are available upon reasonable request. | This has been corrected, emphasizing that: All relevant data are within the manuscript and its supporting information files. Data are available upon reasonable request from the first author | Pg 27 |
| 18. | Overstatement of novelty: The claim that data on STHIs in DM patients in Uganda are “scarce” may be true, but the absence of STHIs limits the novelty of findings | To address this, our manuscript has been reframed to focus on intestinal parasitosis rather than STHIs | Throughout the manuscript |
| 19. | No multivariate analysis was possible, yet the abstract implies assessment of “associated factors.” this is misleading. | With a focus on intestinal parasitosis, factor analysis was possible, and it has been performed | Pg 17-19 |
| 20. | Gastrointestinal symptoms (72.6%) are high, yet only 2% were linked to STHIs this disconnect warrants deeper exploration of alternative causes. | We agree to this. |  |
| 21. | Pet ownership (67.7%) is noted but not discussed as a potential risk factor for non-STH parasites (e.g., Giardia, Hymenolepis). | We have discussed pet ownership as a risk factor for intestinal parasitosis. | Pg 22 |
| 22. | Urban vs. rural residence is reported, but no analysis compares parasitic infection rates by residence, a missed opportunity. | We have performed an analysis to compare parasitic infection by residence and found no association | Pg 17-19 |
| 23. | Fasting blood sugar levels are high (mean 11.9 mmol/L), indicating poor glycemic control, yet no correlation is attempted with parasite presence. | We have performed a correlation for Fasting Blood sugar with intestinal parasites, though it wasn’t found to be significant (p=0.256) | Pg 17 |
| 24. | Discuss public health implications of finding pathogenic non-STH parasites (e.g., E. histolytica, Giardia) in immunocompromised DM patients. | This has been discussed, and we have emphasized the risk of worsening morbidity and mortality in this population. Additionally, implementing screening for these patients generates a direct cost impact on the health sector. | Pg 21 |
| 25. | Reference list includes a 2025 citation (Debash et al., 2025). Verify if this is an in-press or ahead-of-print article; otherwise, it may be inaccurate. | We’ve verified and found that it’s a valid article, published in the BMC Infectious Diseases journal ([https://doi.org/10.1186/s12879-025-10441-4](https://drive.google.com/file/d/1yTRy1FixQGQJCKBaDM2qBw3xMNrwsBi8/view?usp=sharing)) |  |
| 26. | In light of your findings, do you still believe routine STHI screening is warranted for DM patients in this setting, or should screening focus on all intestinal parasites? | Screening should focus on all intestinal parasites and not only STHI as previously thought | Pg 25 |
| **REVIEWER 2 COMMENTS** | | | |
| 1. | Clarify the ethical approval timeline, especially the 2025 amendment date, to avoid confusion or perceived errors. | We received initial approval on 29/09/2024 and started data collection in October 2024. We then made amendments, and the amendment approval was given on 30/05/2025 | Pg 8 |
| 2. | Harmonize the data availability statement across the manuscript and submission form to comply with PLOS ONE policy. | We have harmonized this emphasizing that; All relevant data are within the manuscript and its supporting information files. Data are available upon reasonable request from the first author | Pg 27 |
| 3. | Add a subsection in the Discussion on the possible impact of Uganda’s national deworming programs or improved sanitation on STHI reduction. | With a focus on intestinal parasitosis, our study revealed evidence of poor sanitation (ongoing fecal oral contamination) by the presence of Entamoeba coli. We therefore recommended enhancement of the WASH strategy and screening as preventive strategies to combat this ongoing spread. | Pg 19, 25 |
| 4. | Recommend future studies use triplicate stool sampling and molecular methods to enhance detection sensitivity. | This recommendation has been included | Pg 25 |
| 5. | Improve methodological transparency: Specify how many Kato-Katz thick smears were prepared per sample and who performed microscopy. | This has been addressed.  Two (2) smears were prepared by a laboratory technician and a parasitology professor. | Pg 7 |
| 6. | Consider subgroup analysis of the 39 parasitized vs. 162 non-parasitized patients to identify risk factors for any intestinal parasite. | We have performed this subgroup analysis. | Pg 17-19 |
| 7. | Revise the title and abstract to reflect the actual findings (e.g., “Absence of Soil-Transmitted Helminths but Presence of Other Intestinal Parasites...”). | The title and abstract have been revised | Title page, Pg 1,2 |
| 8. | Why was only one stool sample collected per participant, despite WHO recommendations for multiple samples to improve sensitivity? | We obtained a single stool sample because patients come to the clinic every 1 month and could not meet the 3-day WHO-recommended interval between stool sample collection.  Additionally, most of our participants resided in locations distant from our study site and couldn’t come on 3 separate days to provide samples.  Furthermore, there were limited funds to facilitate the laboratory technician to analyze the 3 samples.  We did not have access to any molecular diagnostics, since they weren’t available in the laboratory at our study site, and there were limited funds to transport and facilitate molecular diagnosis elsewhere. |  |
| 9. | Were any quality control measures (e.g., duplicate readings, expert validation) implemented during microscopic examination? | Quality control measures were implemented. Stool samples that had similar identification numbers were not considered for analysis to avoid duplicates. Every parasite that was not clear to the laboratory technician was proofread and confirmed by the professor of parasitology. |  |
| 10. | How was “uncontrolled diabetes” defined was it based solely on fasting blood sugar, or were HbA1c levels also considered? | Uncontrolled diabetes was defined based on fasting blood sugar, because we could not perform HbA1c, since it was absent at the study site, and needed funds to be done elsewhere, which we didn’t have, as the study was not funded. |  |
| 11. | What is the clinical significance of detecting Candida albicans in stool? Was this considered contamination, colonization, or invasive infection? | There is no clinical significance of finding Candida in stool. We considered it a contamination, and for clarity, we have removed Candida from our results. | All through the manuscript. |
| 12. | Did you consider analyzing associations between parasite presence and diabetes complications (e.g., neuropathy, retinopathy)? | Analysis of associations has been done, and we found no association of intestinal parasites with DM complications (p=0.900) | Pg 17-19 |
| 13. | How generalizable are your findings to rural Ugandan populations, given that over half your sample was urban? | Our findings are not generalizable to the rural population. We have included this in our limitations |  |
| 14. | Were participants screened for HIV or other immunosuppressive conditions that might influence parasite susceptibility? | No, we did not screen the participants for HIV or other immunosuppressive conditions |  |
