# Supplementary material for: Prevalence and factors associated with Intestinal parasitosis among adults with diabetes mellitus attending a tertiary care facility in Northern Uganda: A hospital-based cross-sectional study
Source: PLoS One. 2025 Dec 26;20(12):e0338774. doi: 10.1371/journal.pone.0338774 (PMC12742753; doi:10.1371/journal.pone.0338774)
Supplement: Attachment — Submitted filename: RESPONSE_TO_REVIEWERS_auresp_2.docx [file pone.0338774.s004.docx]

**RESPONSE TO REVIEWERS II**

On behalf of the authors’ team for our study “*Prevalence and factors associated with Intestinal parasitosis among adults with diabetes mellitus attending a tertiary care facility in Northern Uganda: A hospital-based cross-sectional study*”, we sincerely thank the reviewers for the additional feedback and comments on our manuscript. The comments we received have significantly improved the clarity, rigor, and scientific impact of our work. In the table below, we provided a point-by-point response to each comment we received, explaining the revisions made and indicating where the changes were incorporated in the revised manuscript. We believe these changes address all concerns and enhance the paper’s contribution to the field. We look forward to your kind feedback.

| **S/No** | **Comment** | **Response** | **Page in mark-up copy** |
| --- | --- | --- | --- |
| 1 | The abstract contains several grammatical errors and awkward phrases that affect readability. | This has been addressed | Page 1 and 2 |
| 2. | Consistently refer to *E. coli* as a **"non-pathogenic intestinal protozoan"** or **"commensal"** throughout the manuscript. | We have corrected this | Line 35 (Page 2),  Line 180 (Page 14)  Line 206 (Page 17)  Line 265 (Page 20) |
| 3. | The Discussion still contains a long paragraph (pages 58-59, lines 242-261) discussing factors associated with STH and *Strongyloides stercoralis*, which were not found in this study | This has been rectified. |  |
| 4. | In the manuscript (page 18, line 101) and the ethics statement (page 7), the **initial approval date is listed as "29/09/2025"**. This is a clear typo, as data collection started in October 2024. | We have corrected this | Line 122 (Page 6) in the manuscript and also in the ethical statement. |
| 5. | Consider strengthening this point by explicitly stating that the urban skew of the sample limits the applicability of the findings to predominantly rural Northern Uganda. | We have addressed this | Line 256 (Page 20) |
